# Regulation of brachyury by fibroblast growth factor receptor 1 in lung cancer

**DOI:** 10.18632/oncotarget.13547

**Published:** 2016-12-24

**Authors:** Yunping Hu, Xin Feng, Akiva Mintz, W. Jeffrey Petty, Wesley Hsu

**Affiliations:** ^1^ Department of Neurosurgery, Wake Forest School of Medicine, Medical Center Boulevard, Winston-Salem, NC 27157, USA; ^2^ Department of Otolaryngology, Wake Forest School of Medicine, Medical Center Boulevard, Winston-Salem, NC 27157, USA; ^3^ Department of Radiology, Wake Forest School of Medicine, Medical Center Boulevard, Winston-Salem, NC 27157, USA; ^4^ Department of Hematology and Oncology, Wake Forest School of Medicine, Medical Center Boulevard, Winston-Salem, NC 27157, USA

**Keywords:** fibroblast growth factor receptor 1, mitogen-activated protein kinase, extracellular signal-regulated kinase, brachyury, lung cancer

## Abstract

Recent evidence suggests that T-box transcription factor brachyury plays an important role in lung cancer development and progression. However, the mechanisms underlying brachyury-driven cellular processes remain unclear. Here we found that fibroblast growth factor receptor 1/mitogen-activated protein kinase (FGFR1/MAPK) signaling regulated brachyury in lung cancer. Analysis of FGFR1-4 and brachyury expression in human lung tumor tissue and cell lines found that only expression of FGFR1 was positively correlated with brachyury expression. Specific knockdown of FGFR1 by siRNA suppressed brachyury expression and epithelial–mesenchymal transition (EMT) (upregulation of E-cadherin and β-catenin and downregulation of Snail and fibronectin), whereas forced overexpression of FGFR1 induced brachyury expression and promoted EMT in lung cancer cells. Activation of fibroblast growth factor (FGF)/FGFR1 signaling promoted phosphorylated MAPK extracellular signal-regulated kinase (ERK) 1/2 translocation from cytoplasm to nucleus, upregulated brachyury expression, and increased cell growth and invasion. In addition, human lung cancer cells with higher brachyury expression were more sensitive to inhibitors targeting FGFR1/MAPK pathway. These findings suggest that FGFR1/MAPK may be important for brachyury activation in lung cancer, and this pathway may be an appealing therapeutic target for a subset of brachyury-driven lung cancer.

## INTRODUCTION

Lung cancer is the most common cause of cancer death worldwide. Previous studies on molecular profiling have defined potential subsets of lung cancer patients [1–3], which in turn has resulted in new molecularly targeted therapies [4]. Many of these therapies aim at biomarkers that are overexpressed in cancers and are involved in cell growth, proliferation, migration, and survival [5]. However, the major issue of targeted therapy is the occurrence of drug resistance. [6]. Therefore, current efforts have been made to identify novel biomarkers and its potential molecular mechanisms underlying resistance to targeted therapies.

Recent studies have shown that the T-box transcription factor brachyury, an embryonic determinant of endodermal and mesodermal lineage differentiation during embryonic development [7], plays a role in initiating the processes that lead to the growth and spread of cancer [8–12]. Brachyury expression has been detected in 41% of primary lung tumor tissues, including 48% of adenocarcinomas and 25% of squamous carcinomas [9]. It is also a significant predictor of poor prognosis in primary lung carcinoma [10]. Functional studies further demonstrated that inhibition of brachyury by shRNA leads to downregulation of mesenchymal markers, inhibition of H460 lung cancer cell migration and invasion, and decreased ability of tumor cells to form distant metastases *in vivo* [13]. Brachyury also blocks lung cancer cell cycle progression and mediates tumor resistance to various conventional chemotherapies and radiation [14]. Although these studies suggested that brachyury facilitates lung cancer development and progression, the particular mechanisms underlying brachyury activation in lung cancer remain unknown.

In the early embryo, brachyury expression requires the activation of fibroblast growth factor (FGF) and their receptor (FGFR) [15, 16], and a high-level of FGF/FGFR signaling maintains brachyury [17]. Activation of FGF/FGFR signaling initiates several intracellular signaling, including the mitogen-activated protein kinase (MAPK) cascade, which is an essential pathway during embryonic development [18]. The activated MAPK extracellular signal-regulated kinase (ERK) translocates to the nucleus and activates transcription factors to induce abnormal gene expression and promote growth, differentiation and survival [19, 20]. Previous studies showed that FGFR/ERK mediates mesodermal induction by brachyury [21, 22], whereas blocking FGFR/ERK signaling results in a loss of brachyury expression and suppresses FGF-induced mesoderm formation and angiogenesis [23]. Genetic alterations in FGFR including gene amplifications, somatic missense mutations and chromosomal translocations which lead to overexpression and/or constitutive activation of FGFR have also been found in lung cancer [24, 25] and the suppression of FGFR signaling significantly inhibits tumor growth and survival [25, 26]. Despite the observations of abnormal FGFR expression in lung cancer, it remains unclear whether such receptor alternation drives specific molecularly defined subsets of lung cancer. An understanding of the role of FGFR signaling in brachyury activation may elucidate a novel therapeutic target for lung cancer initiation and progression.

In the present study, we examine whether FGFR modulate cellular tyrosine phosphorylation and activate brachyury to promote lung cancer progression. Firstly, we analyze FGFR and brachyury expressions in human lung tumor tissues and cell lines to investigate their associations. We then evaluated the impacts of FGFR inputs or knockdown on brachyury expression in lung cancer cells following a biological function studies including the change of epithelial–mesenchymal transition (EMT), cell/tumor growth and cell invasion. Our study demonstrates that FGFR1/MAPK signaling potentially contributes to brachyury activation and suggests that targeting FGFR1/MAPK may represent a useful strategy to suppress brachyury-driven lung cancer progression.

## RESULTS

### Brachyury expression is highly associated with FGFR expression in human lung tumor tissues and cells lines

**Figure 1:**
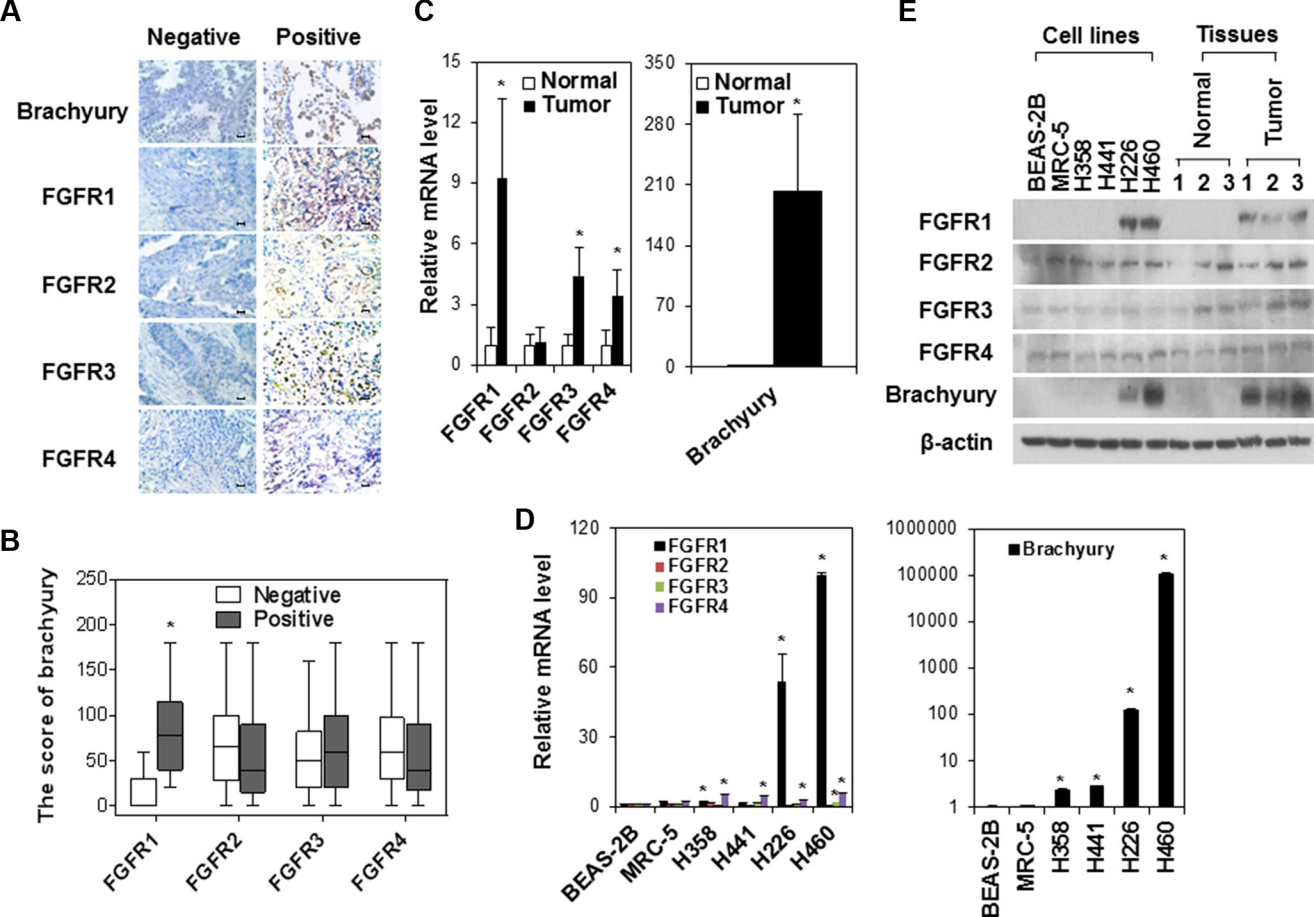
The association of FGFR and brachyury expressions in human lung tumor tissues and cell lines (**A**) Representative IHC staining of brachyury and FGFR1-4 in human lung tumor tissue array samples (*n* = 61). Negative: no staining or < 5% staining of tumor cells. Positive: ≥ 5% staining of tumor cells. Scale bar = 100 μm. (**B**) The score of brachyury in human lung tumor tissue array samples with FGFR1-4 positive staining. * *P* < 0.05, vs negative. The boxplots indicate the minimum, the first quartile, median, third quartile, and maximum. *P < 0.05, vs negative. (**C**) Quantitative RT-PCR analysis of FGFR1-4 and brachyury mRNA expression in human lung adenocarcinoma tissues and adjacent normal lung tissues. Data were presented as mean ± SD (*n* = 25). *P < 0.05, vs normal tissues. (**D**) Quantitative RT-PCR analysis of FGFR1-4 and brachyury expression in normal lung cell lines (BEAS-2B and MRC-5), non-metastatic H358 and H441 tumor cell lines, and metastatic H226 and H460 cell lines. Data were presented as mean ± SD (*n* = 3). *P < 0.05 vs BEAS-2B. (**E**) Representative Western blot analysis for FGFR1-4 and brachyury expression in normal cell line BEAS-2B and MRC-5, lung cancer cell line H358, H441, H226 and H460, and human lung adenocarcinoma tissues and adjacent normal lung tissues (No. 1, 2, 3) from three experiments with similar results.

To investigate the associations between brachyury and FGFR in lung cancer, we measured brachyury and FGFR1-4 expressions in human lung tumor tissues and cells lines. IHC staining for paraffin-embedded human lung tumor tissue array found that most tumor tissue samples had immunoreactivities. The representative IHC staining for FGFR 1-4 and brachyury are showed in Figure 1A. The percentages of positive staining for at least one FGFR or brachyury were 66% (FGFR1), 57% (FGFR2), 64% (FGFR3), 61% (FGFR4) and 45% (brachyury), respectively. Further analysis disclosed that tumor tissues with FGFR1 immunoreactivity had significantly higher score of brachyury staining (Figure 1B). Considering that small cores used to construct a tumor tissue array may not accurately represent characteristics of the whole tissue specimen [27] and a semi-quantitative IHC scoring could introduce potential bias into interpretation of results [28], we further collected whole tumor tissue sections to quantitatively evaluate FGFR gene expression profile. Comparisons of brachyury and FGFR mRNA levels in paired lung tumor and adjacent normal tissues demonstrated that tumor tissues had significantly higher expressions of FGFR1, 3 and 4 and brachyury than normal tissues adjacent to tumor (Figure 1C). Spearman’s correlation analysis showed that brachyury mRNA level was significantly correlated with FGFR1, FGFR3 and FGFR4 mRNA levels in lung tumor tissues but not in adjacent normal tissues. Similar association between FGFR1, FGFR3, FGFR4 and brachyury gene expression was also observed in lung cancer cell lines (Figure 1D). In addition, chemotherapy-insensitive/metastatic cell lines H226 and H460 and human lung tumor tissues had higher FGFR1and brachyury protein expressionthan chemotherapy-sensitive/non-metastatic cell lines H358 and H441 and normal tissues adjacent to tumor (Figure 1E). Taken together, our observation of the endogenous FGFR upregulation correlating with brachyury in lung cancer suggest that abnormal overexpression of FGFR may coordinate the activation of brachyury to promote tumor progression.

### Brachyury activation is regulated by FGFR1 in lung cancer

**Figure 2:**
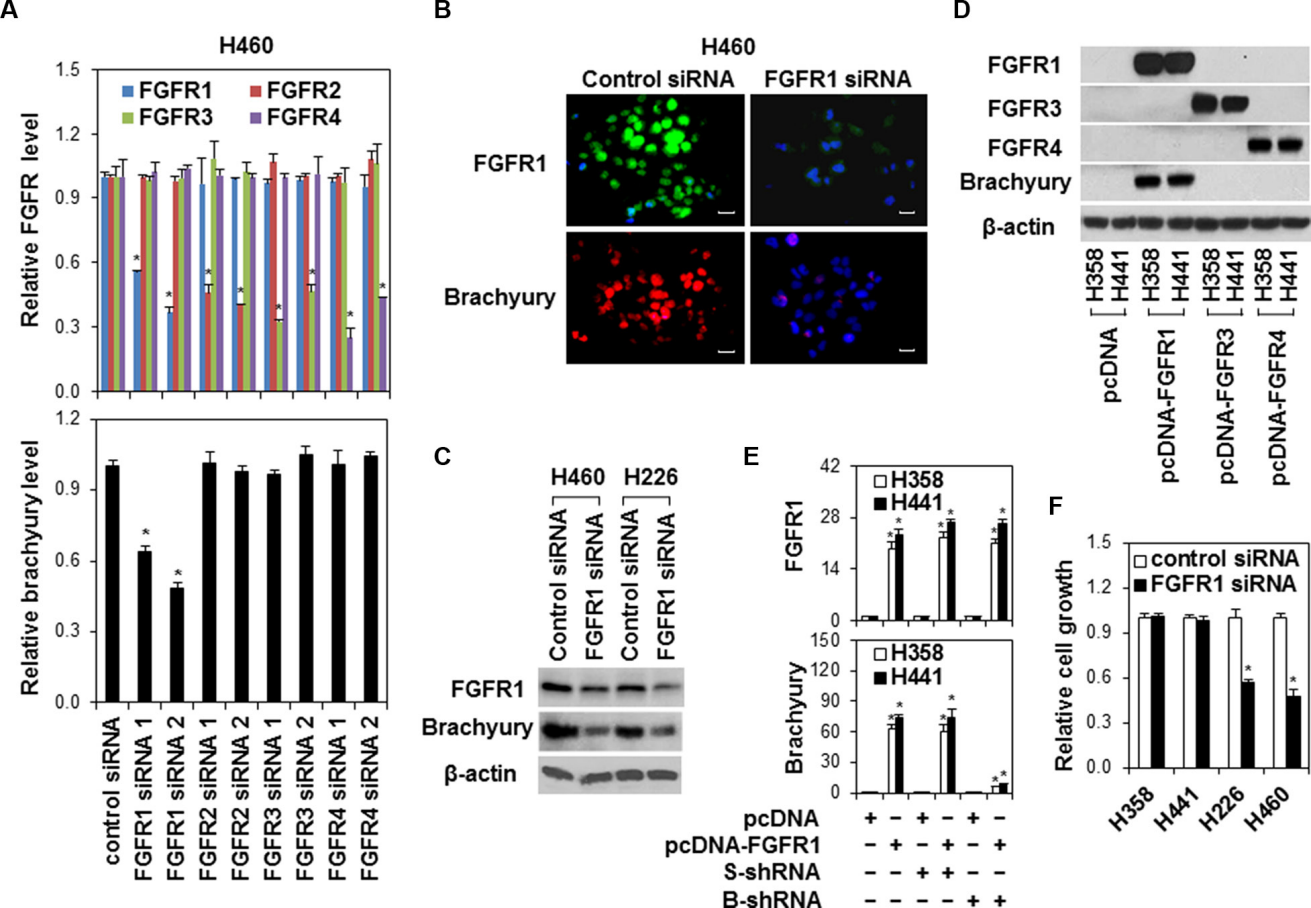
The regulation of brachyury by FGFR1 in lung cancer cells (**A**) H460 cells were transfected with control siRNA or FGFR 1-4 siRNAs for 24 h. Quantitative RT-PCR assay was used to measure FGFR1-4 and brachyury expressions. Data were presented as mean ± SD (*n* = 4). *P < 0.05, vs control siRNA. (**B**) H460 cells were transfected with control siRNA or FGFR 1 siRNA. Cells were fixed and immunofluorescence stained for FGFR1 with anti-FGFR1 (green) or for brachyury with anti-brachyury (red). Nuclei were counter-stained with DAPI (blue). Scale bars = 50 μm. Representative images from three independent experiments are shown. (**C**) H460 and H226 cells were transfected with control siRNA or FGFR 1 siRNA (FGFR1 siRNA 2) for 48 h. Cell protein extracts were used for Western Blot analysis of FGFR1 and brachyury. Representative bands from three independent experiments with similar results are shown. (**D**) H358 and H441 cells were stably transfected with pcDNA empty, pcDNA-FGFR1, pcDNA-FGFR3 or pcDNA-FGFR4 vector. Cell protein extracts from stably transfected clones were used for Western Blot analysis of FGFR 1-4 and brachyury expression. Representative bands from three independent experiments with similar results are shown. (**E**) H358 and H441 cells stably expressing pcDNA or pcDNA-FGFR1 were transfected with scramble shRNA (S-shRNA) or brachyury shRNA (B-shRNA) for 24 h. FGFR1 and brachyury gene expressions were measured by quantitative RT-PCR. Data were presented as mean SD (*n* = 4). *P < 0.05, vs pcDNA; ^#^*P* < 0.05, vs pcDNA-FGFR1. (**F**) H358, H441, H226 and H460 cells were transfected with control siRNA or FGFR1 siRNA (FGFR1 siRNA 2) for 48 h. Cell growth was measured by MTS. Data were presented as mean ± SD (*n* = 5). *P < 0.05, vs control siRNA.

To explore the specific function of FGFR in brachyury activation in lung cancer, we silenced FGFR expressions by siRNAs in lung cancer cell line H460, which has higher endogenous FGFR and brachyury expressions (Figure 1D and 1E). We found that only FGFR1 inhibition led to suppression of brachyury in H460 cells (Figure 2A). Western blot and immunostaining assays further confirmed the inhibitory effect of FGFR1 silence on brachyury expression (Figure 2B and 2C). Considering the lower endogenous expression of FGFR1, FGFR3 and FGFR4 in human lung cancer cell line H441 and H358, we forced expression of full-length FGFR1, FGFR3 and FGFR4 in H441 cells. Only overexpression of FGFR1 induced brachyury expression (Figure 2D and 2E), and this induction of brachyury expression was blocked by shRNA mediated brachyury inhibition (Figure 2E). In addition, silencing of FGFR1 by FGFR1 siRNA only inhibited the growth of cells (H226 and H460) with higher brachyury expression (Figure 2F). These data imply that FGFR1 is a potential modulating factor of brachyury activation in lung cancer cells.

### FGFR1 manipulates brachyury through MAPK signaling

**Figure 3:**
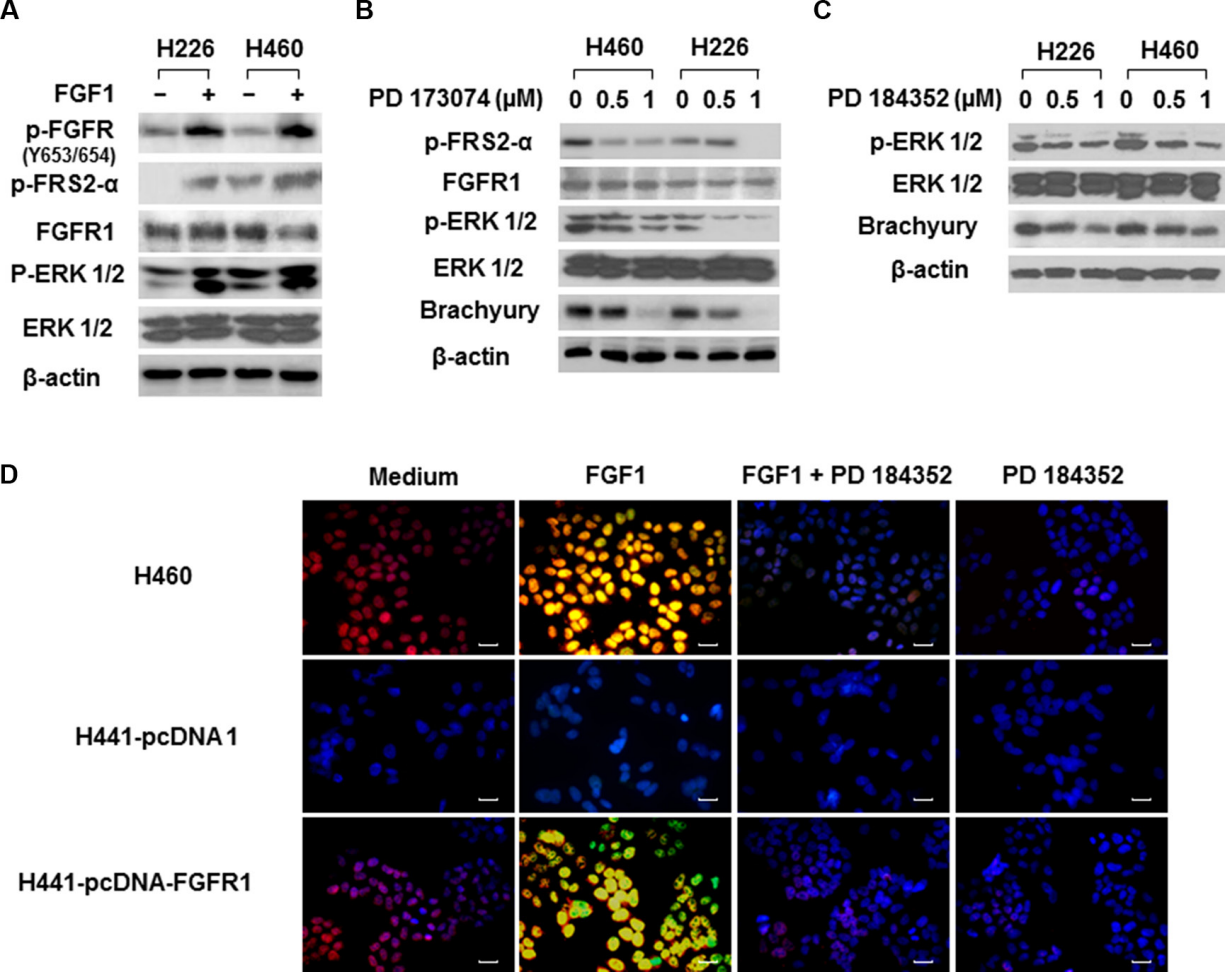
The MAPK-mediated regulation of FGFR1 on brachyury in lung cancer cells (**A**) H226 and H460 cells were seeded in 6-well plates at density of 2 × 10^5^ cells per well for 24 h, then cells were starved overnight and treated with FGF1 (100 ng/ml) for 10 min. Cell protein extracts were used for Western Blot analysis of FGFR1, FGFR phosphorylations (p-FGFR and p-FRS2), ERK and ERK phosphorylation (p-ERK 1/2). Representative bands from three independent experiments with similar results are shown. (**B** and **C**) H460 and H226 cells were treated with FGFR inhibitor (PD 173074) and MAPK kinase inhibitor (PD 184352) at the indicated doses for 48 h. Cell protein extracts were used for Western Blot analysis of p-FSR2-α (B), FGFR1 (B), ERK (B & C), p-ERK 1/2 (B and C) and brachyury (B and C). Representative bands from three independent experiments with similar results are shown. (**D**) H460 cells or H441 cells stably expressing pcDNA or pcDNA-FGFR1 were seeded in 24-well plates at density of 2 × 10^4^ cells per well for 24 h, then cells were starved overnight and treated with FGF1 (10 ng/ml) for 24 h followed by PD 184352 treatment (1 μM) for 1 h. Cells were fixed and double immunofluorescence stained for p-ERK 1/2 with anti-p-ERK 1/2 (green) and for brachyury with anti-brachyury (red). Nuclei were counter-stained with DAPI (blue). Yellow color indicated the co-localization of p-ERK 1/2 and brachyury. Scale bars = 50 μm. Representative images from three independent experiments are shown.

Our Western blot assay showed that FGF1 stimulation induced autophosphorylation of FGFR1 (phosphorylation of FRS2-α Tyr196), and triggered ERK phosphorylation (Figure 3A). Inhibition of FGFR1/ MAPK ERK signaling by the FGFR inhibitor PD 173074 and MAPK kinase inhibitor PD 184352 significantly suppressed brachyury expression in H460 and H226 cells in a dose-dependent manner (Figure 3B and 3C). Immunofluorescence staining further demonstrated that the activation of FGFR1 by recombinant human FGF1 promoted phosphorylated ERK 1/2 translocation from cytoplasm to nucleus and increased brachyury expression in H460 cells (Figure 3D). The forced overexpression of FGFR1 increased the sensitivity of H441 cells to FGF1-triggered effects on ERK 1/2 phosphorylation and brachyury expressions (Figure 3D). In addition, FGF1-mediated activation of ERK/brachyury was blocked by PD 184352 (Figure 3D). These data demonstrate that MAPK mediates the regulation of FGFR1 in brachyury activation.

### FGFR1 modulates brachyury-driven EMT via MAPK

**Figure 4:**
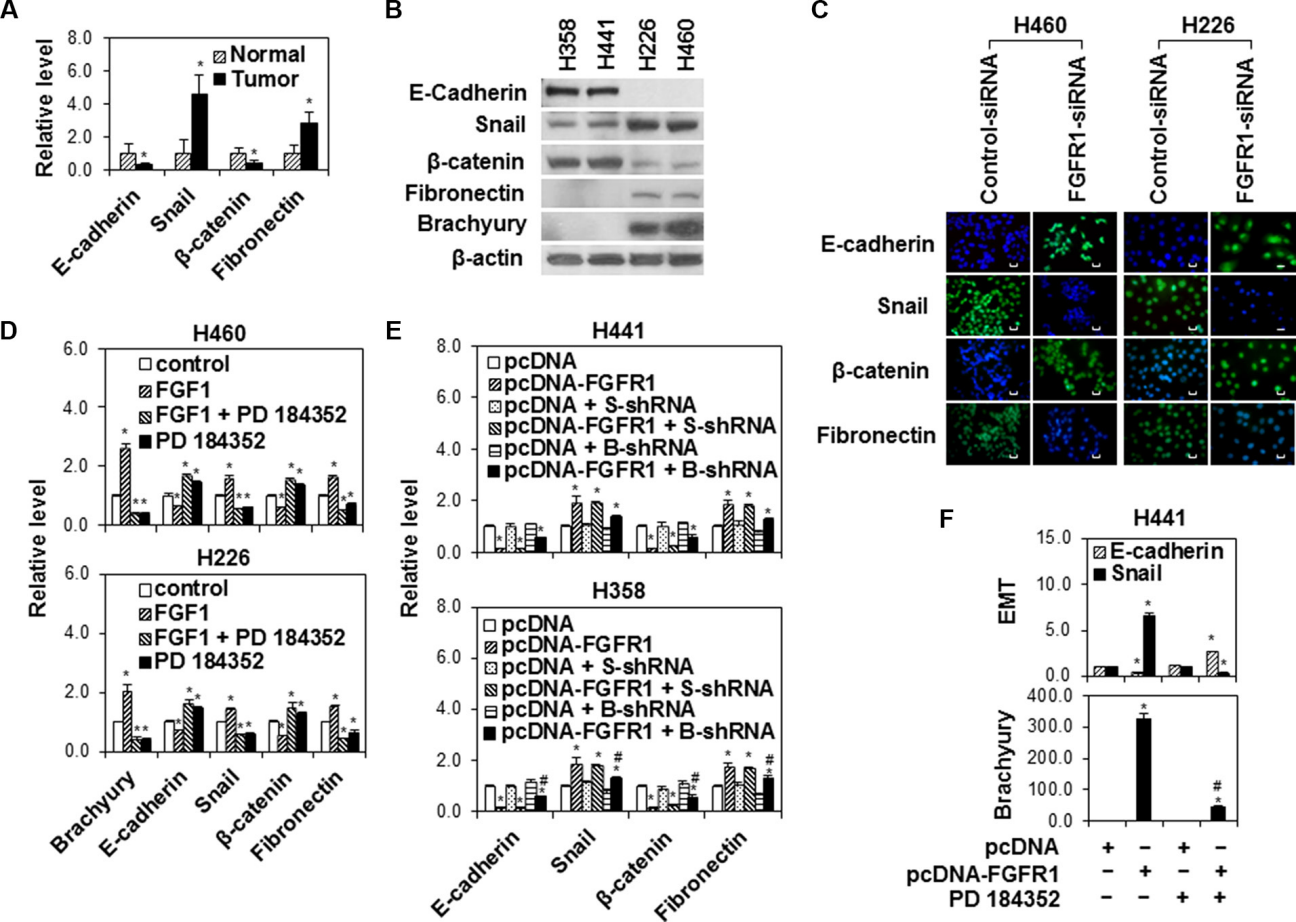
The modulation of brachyury-driven EMT by FGFR1/MAPK signaling in lung cancer cells (**A**) Quantitative RT-PCR analysis of E-cadherin, Snail, β-catenin and fibronectin mRNA expression in human lung adenocarcinoma tissues and adjacent normal lung tissues. Data were presented as mean ± SD (*n* = 25). *P < 0.05, vs normal tissues. (**B**) H358, H441, H226 and H460 cells were seeded in 6-well plates at density of 2 × 10^5^ cells per well for 24 h. Cell protein extracts were used for Western Blot analysis of E-cadherin, Snail, β-catenin, fibronectin and brachyury. Representative bands from three independent experiments with similar results are shown. (**C**) Immunofluorescence staining for E-cadherin, Snail, β-catenin and fibronectin (green) in H460 and H226 cells after transfection with control siRNA or FGFR1 siRNA (FGFR1 siRNA 2) for 48 h. Nuclei was counter-stained with DAPI (blue). Scale bars = 50 μm. Representative images from three independent experiments are shown. (**D**) H460 and H226 cells were seeded in 6-well plates at density of 2 × 10^5^ cells per well for 24 h, then cells were starved overnight and treated with FGF1 (10 ng/ml) for 24 h followed by PD 184352 treatment (1 μM) for 1 h. Quantitative RT-PCR assay was used to measure brachyury, E-cadherin, Snail β-catenin and fibronectin expressions. *P < 0.05, vs control (untreated). (**E**) H441 and H358 cells stably expressing pcDNA or pcDNA -FGFR1 were transfected with scramble shRNA (S-shRNA) or brachyury shRNA (B-shRNA) for 24 h. Quantitative RT-PCR assay was used to measure E-cadherin, Snail, β-catenin and fibronectin expressions. Data were presented as mean ± SD (*n* = 3). *P < 0.05, vs pcDNA alone; ^#^*P* < 0.05, vs pcDNA-FGFR1 alone. (**F**) H441 cells stably expressing pcDNA or pcDNA-FGFR1 were treated with PD 184352 (1 μM) for 24 h. E-cadherin, Snail and brachyury expressions were measured by Quantitative RT-PCR assay. Data were presented as mean ± SD (*n* = 3). *P* < 0.05, vs pcDNA alone.

EMT induction contributes to tumor progression and metastasis [29]. Previous studies have shown that brachyury regulates EMT in lung cancer [13]. However, the mechanism by which brachyury initiates EMT in lung cancer is unclear. In this study, we examined expressions of EMT biomarkers including E-cadherin, Snail, β-catenin, and fibronectin in 25 paired lung tumor and adjacent normal tissues by RT-PCR and found that tumor tissues had lower E-cadherin and β-catenin expressions and higher Snail and fibronectin expressions than adjacent normal tissues (Figure 4A). Further Western blot analysis of EMT in lung cancer cell lines also demonstrated that H358 and H441 cells with lower endogenous FGFR and brachyury had higher E-cadherin and β-catenin expressions and lower Snail and fibronectin expressions than H226 and H460 cells with higher endogenous FGFR and brachyury (Figure 4B). The silence of FGFR1 by siRNA significantly upregulated the levels of E-cadherin and β-catenin and downregulated the levels of Snail and fibronectin in H460 and H226 cells (Figure 4C). In addition, FGF1 stimulation decreased the levels of E-cadherin and β-catenin and increased the levels of Snail and fibronectin, whereas the MAPK kinase inhibitor PD 184352 reversed FGF1-induced effects on E-cadherin, Snail, β-catenin and fibronectin expressions in H460 and H226 (Figure 4D). Forced FGFR1 also decreased E-cadherin andβ-catenin levels and increased Snail and fibronectin levels, whereas brachyury knockdown by shRNA or PD 184352 reduced the impact of forced FGFR1 on EMT (Figure 4E and 4F). These data suggest an important regulatory role of FGFR1/MAPK signaling in brachyury-initiated EMT in lung cancer cells.

### FGFR1/MAPK signaling controls brachyury-driven lung cancer cell/tumor growth and cell invasion

**Figure 5:**
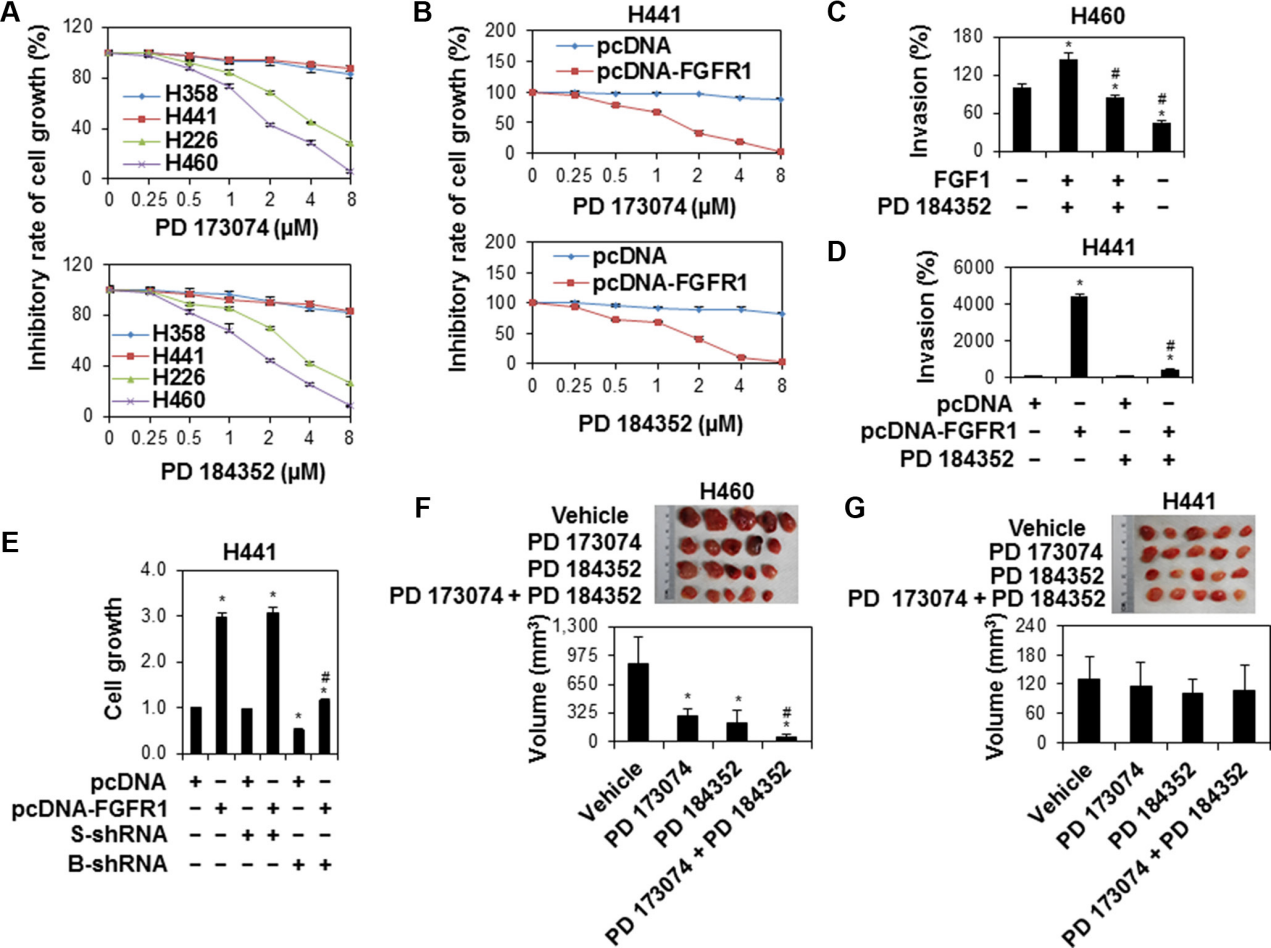
The suppressed lung cancer cell growth and invasion by inhibition of FGFR/MAPK/brachyury signaling (**A**) Human lung cancer cell lines H358, H441, H226 and H460 were treated with PD 173074 or PD 184352 at the indicated doses for 48 h. Cell growth was measured by MTS. Data were presented as mean ± SD (*n* = 5). (**B**) H441 cells stably expressing pcDNA or pcDNA-FGFR1 were treated with PD 173074 or PD 184352 at the indicated doses for 48 h. Cell growth was measured by MTS. Data were presented as mean ± SD (*n* = 4). *P < 0.05, vs pcDNA at the same dose of treatment. (**C**) H460 cells were pretreated with MAPK inhibitor, PD 184352 (1 μM) for 1 h after starving overnight, following an incubation with FGF1 (10 ng/ml) for 24 h. Cell invasion was measured by BD matrigel invasion assay. The y-axis represents a percent change over serum-free control. Error bars indicate SEM (*n* = 4). (**D**) H441 cells stably expressing pcDNA or pcDNA-FGFR1 were treated with PD 184352 (1 μM) for 48 h. Cell invasion was measured by BD matrigel invasion assay. *P < 0.05, vs pcDNA at the same dose of treatment. ^#^*P* < 0.05, vs FGF1+ PD 184352. Data were presented as mean ± SEM (*n* = 4). for cells stably expressing pcDNA alone). (**E**) H441 cells stably expressing pcDNA or pcDNA-FGFR1 were transfected with scramble shRNA or brachyury shRNA for 48 h. Cell growth was measured by MTS. Data were presented as mean ± SD (*n* = 4). *P < 0.05, vs control (untreated for cells stably expressing pcDNA alone). ^#^*P* < 0.05, vs PD 184352. (**F** and **G**) Antitumor activity of PD 173074 or/and PD 184352 in lung cell line xenografts. H460 (F) and H441 (G) cells were implanted into the flank of nude mice. PD 173074 or/and PD 184352 or vehicle (buffer) i.p treatment were administered daily (*n* = 5 per group). Tumor volumes were measured at 12 d after initiation of treatments. Top: Samples of lung tumors in different treatment groups. Bottom: The volume of tumor which regressed with PD 173074 or/and PD 184352 treatment. Data were presented as mean ± SD (*n* = 5). *P < 0.05 vs vehicle. ^#^*P* < 0.05, vs PD 173074 or PD 184352 alone.

To test the biological function of FGFR1/MAPK in brachyury-driven lung cancer cell/tumor growth and invasion, we examined the response of lung cancer cell lines with differential brachyury expressions to PD 173074 and PD 184352 and found that cell lines H226 and H460 with endogenously higher brachyury expression were more susceptible to treatment by PD 173074 or PD 184352 (Figure 5A). Forced overexpression of FGFR1 in H441 also increased the sensitivity of cells to these inhibitors (Figure 5B). Activation of FGF/FGFR1 pathway by FGF1 stimulation (Figure 5C) or forced overexpression of FGFR1 (Figure 5D) increased H441 cell invasion, which was abrogated by PD 184352. In addition, knockdown of brachyury by shRNA significantly reduced the effects of forced FGFR1-promoted cell growth (Figure 5E). Furthermore, our *in vivo* study demonstrated that tumor growth was faster in H460 cells-bearing mice than that in H441 cells-bearing mice. In addition, PD 173074 and PD 184352 as a single agent inhibited tumor growth in H460 cells-bearing mice but had no impact on H441 cells-bearing mice. Moreover, the combination of PD 173074 and PD 184352 was significantly better at impairing tumor growth compared with either agent alone (Figure 5F and 5G). These findings suggest that FGFR1/MAPK signaling directs brachyury-driven lung cancer cell progression.

## DISCUSSION

Our present study showed that higher FGFR expressions in human lung tumor tissues and cell lines are positively associated with higher brachyury expression. Forced and silenced expression of FGFR demonstrated the potential function of specific FGFR1 signaling in activating brachyury. Further upregulation and downreguation of FGFR1 signaling revealed that FGFR1 is linked to a mechanism triggering MAPK/ERK phosphorylation and translocation from cytoplasm to nucleus, which appears to be necessary for brachyury activation and is also important for facilitating EMT, cell/tumor growth and invasion of lung cancer. These data indicate that MAPK-mediated FGFR1 signaling plays an important role in the regulation of brachyury. Therefore, targeted inhibition of FGFR1 signaling molecules may inhibit tumor progression in a subset of brachyury-driven lung cancer.

The FGFR constitute a family of four tyrosine kinase receptors, FGFR1 through FGFR4, which mediate cellular signaling after binding to their high-affinity FGF ligands. FGFR regulate a variety of cellular functions, including embryogenesis and adult tissue homeostasis [30, 31]. The overexpression of FGFR and abnormalities in the FGFR signaling axis has also been observed in many cancers including lung cancer [25, 32]. However, the significance of FGFR expression is unclear. Our previous study has showed that FGFR regulates brachyury expression in chordoma [33]. We also found that chordoma and lung cancer have different expression profiles of FGFR, and these profiles may exert distinct effects on brachyury. It is possible that other tumor subtypes will have their own FGFR expression patters, and these expression patterns may have distinct effects on brachyury. In the present study, we have tested the role of FGFR in brachyury activation and found a significant differential association between FGFR subtype and brachyury expressions at protein and gene levels. FGFR1 protein and gene expressions in human lung tumor tissue array are related to relevant brachyury protein and expressions, whereas FGFR3 and FGFR4 expression in paired lung tumor tissues and cell lines is linked to brachyury expression only at gene level (Figure 1B, and 1D). Further functional analysis by silencing or forced FGFR expression at gene and protein levels has disclosed the specific regulation of brachyury by FGFR1 (Figure 2). These data imply that FGFR1 plays a potential driving role in brachyury activation and the abnormal activation or upregulation of FGFR1 may be a predictor of dependency of the cells on brachyury signaling in lung cancer.

FGF/FGFR signaling can activate a number of downstream signaling cascades such as MAPK signaling. The activation of FGFR/MAPK kinase is necessary for the induction of mesoderm in Xenopus embryos and mammals as shown by expression of mesodermal markers brachyury [21, 22, 34]. FGFR/MAPK signaling pathway is also commonly activated in cancers and represents an attractive target for molecular therapy of cancer. Our results indicate that FGF1 or overexpression of FGFR1 induces MAPK/ERK phosphorylation, whereas blocking FGFR1/MAPK signaling by specific inhibitors results in a significant suppression of brachyury. Double immunofluorescence staining demonstrates that activation of FGF1/FGFR1 signaling promotes ERK phosphorylation in the nucleus followed by transcriptional activation of brachyury, implying that the FGF1/FGFR1-induced ERK phosphorylation in the nucleus may change the transcriptional level of brachyury. In addition, the inhibition of MAPK/ERK potentially suppresses FGF1/FGFR1-activated brachyury expression, suggesting that MAPK mediates FGF/FGFR1-promoted brachyury activation.

EMT has been indicated to be involved in cancer growth, metastasis, and drug resistance [29, 35]. Recently, brachyury has been identified as a regulator of EMT in lung cancer. Forced brachyury expression in A549 cells, which does not normally express brachyury, results in changes characteristic of EMT, including increased expressions of N-cadherin and vimentin and decreased expressions of E-cadherin, whereas the blockage of brachyury upregulates plakoglobin expression and downregulates the levels of snail, slug and fibronectin [13]. In addition, previous studies also have demonstrated that FGF/FGFR1 signaling is involved in EMT during development [16] and in models of cancer [36–38]. In the present study, we found that specific knockdown of FGFR1 represses Snail and fibronectin and induces E-cadherin and β-catenin, whereas activation of FGF/FGFR1 signaling promotes this EMT process which can be reversed by MAPK kinase inhibition and brachyury knockdown (Figure 4C–4F). Taken together, these data demonstrate an important regulatory role of FGF/FGFR1 signaling in brachyury-driven EMT through MAPK.

Recent large-scale genomic studies have revealed the genetic landscape of different lung cancer subtypes [1, 2] which provide groundwork for the development of personalized medicine [39]. Identification of genetic abnormalities in FGFR in lung cancer has generated immense interest in targeting FGFR in the clinic [3, 4]. However, it’s currently unclear how to select patients with good response to FGFR-targeted therapy, and preclinical data suggest that additional biomarkers of FGFR pathway activation may be required [40, 41]. Our present study have found that human lung cancer cells in which brachyury is upregulated are more sensitive to inhibitors PD 173074 or PD 184352 targeting FGFR1/MAPK pathway, whereas FGF/FGFR1 activation-promoted lung cancer cell growth and invasion is potentially abrogated by inhibition of MAPK and brachyury, respectively (Figure 5). These findings suggest that a novel strategy of targeting FGFR1/MAPK for treatment of the subset of brachyury-driven lung cancer would be more effective.

In conclusion, our data provide strong evidence that FGFR1 signaling plays an important role in regulating brachyury-driven lung cancer cellular processes by MAPK. Elucidation of this mechanism offers opportunities for application of novel chemotherapeutic strategies against lung cancer that target FGFR1/MAPK/brachyury pathway.

## MATERIALS AND METHODS

### Reagents and antibodies

PD 184352 (an MAPK kinase inhibitor) was purchased from Santa Cruz Biotechnology, Inc (Santa Cruz, CA). PD 173074 (a FGFR inhibitor) was from Cayman Chemical Company (Ann Arbor, MI). Human recombinant FGF1 was purchased from Life Technologies (Grand Island, NY). G418 was from Invivogen (San Diego, CA). Protease inhibitor cocktail (Complete^™^, EDTA-free) and phosphatase inhibitor cocktail (PhosStop) were obtained from Roche Diagnostics (Indianapolis, IN). RPMI 1640 medium was purchased from Thermo Fisher Scientific (Grand Island, NY). X-tremeGENE HP DNA Transfection Reagent was from Roche (Indianapolis, IN). CellTiter 96^®^ AQueous One solution Assay was from Promega (Madison, WI). The antibodies for ERK 1/2, phosphorylated ERK 1/2 (T202/Y204), phosphorylated FGFR (Tyr653/654) and Phosphorylated FGFR substrate 2 (FRS2)-α (Tyr196) were purchased from Cell Signaling Technology (Danvers, MA). The FGFR2 antibody was purchased from GeneTex (San Antonio, Texas). The goat anti-rabbit IgG-HRP, the donkey anti-goat IgG-HRP and antibodies for FGFR1, FGFR3, FGFR4, brachyury, β-catenin, fibronectin, E-cadherin and Snail were purchased from Santa Cruz Biotechnology, Inc (Santa Cruz, CA). Alexa Fluor^®^ 488 AffiniPure Fab fragment goat anti-rabbit IgG (H+L) and Rhodamine Red^™^-X (RRX) AffiniPure F (ab′)_2_ fragment donkey anti-goat IgG (H+L) were from Jackson ImmunoResearch Laboratories (West Grove, PA).

### Cell culture and transfection

Human lung normal cell lines BEAS-2B and MRC-5 and cancer cell lines H358, H441, H226 and H460 were purchased from the American Type Culture Collection (Manassas, VA) and cultured in RPMI 1640 (Thermo Fisher Scientific, Grand Island, NY) supplemented with 10% fetal bovine serum (FBS) at 37°C in 5% CO2. Transfections of plasmids and validated siRNA specifically targeting human FGFR (Ambion, Carlsbad, CA) were performed using X-tremeGENE HP DNA Transfection Reagent and Lipofectamine^®^ 2000, respectively, following the manufacturers’ instructions. The plasmid expressing brachyury-specific shRNA (Johns Hopkins University High Throughput Biology Center) or a control scramble shRNA (Addgene) were generated by inserting the reported sequences into the pLKO.1 vector. Plasmids empty pcDNA3.1 (pcDNA) and pcDNA3.1-FGFR1 (pcDNA-FGFR1) containing full-length human FGFR1 and plasmids pcDNA3.1- FGFR3 (pcDNA-FGFR3) and pcDNA 3.1-FGFR4 (pcDNA-FGFR4) containing full-length human FGFR3 and FGFR4 were kindly gifted by Prof. Michal K. Stachowiak at State University of New York at Buffalo and Prof. Daniel J. Donoghue at University of California, respectively. For stable transfection, brachyury shRNA plasmid or scramble shRNA plasmid was transfected into H441 for 48 h, then G418 (1000 μg/ml) was added and the cells were selected for 14 days. The surviving cells were selected, expanded and screened by Western blotting. Cell population showing the overexpression of transfected genes was subsequently subjected to single cell clonal expansion and maintained in G418 (300 μg/ml). For transient transfection, FGFR siRNAs and control siRNA were transfected into H460 and H226 cells, whereas plasmid brachyury shRNA and scramble shRNA was transfected into H441 and H358 stable transfected cells for 24–48 h.

### Tissue collection and processing

Twenty-five human lung adenocarcinoma tissues paired with their corresponding adjacent normal tissue were obtained from the Advanced Human Tissue Bank at Wake Forest Health Sciences with institutional review board approval. Information concerning the patients’ primary diagnoses was collected; however, no patient identifiers were obtained. Total RNA was isolated from 50 to 100 mg of above frozen tissues using Trizol (Life Technologies Chemical, Ann Arbor, MI) following the manufacturer’s instructions and stored at −80°C until PCR amplification.

### Immunohistochemistry (IHC) and scoring of human lung tumor tissue arrays

The paraffin-embedded human lung tumor tissue array containing 61 different lung tumors (22 adenocarcinoma, 28 squamous cell carcinoma, 6 alveolar carcinoma, 1 large cell carcinoma and 4 small cell carcinoma) were purchased from BioChain Institute, Inc. (Newark, CA). Tissue sections were 5 μm in thickness and mounted on positively charged glass slides. Slides were deparaffinized and rehydrated. Endogenous peroxidase activity was blocked using 3% hydrogen peroxide for 10 min. Heated-antigen retrieval was performed at 10 mM sodium citrate buffer (pH: 6.0) for 10 minutes. IHC was carried out using a Vectastain Elite ABC kit (Burlingame, CA), as per the manufacturer’s instructions. Slides were blocked and incubated with primary antibodies for FGFR1-4 (1:100) and brachyury (1:200) overnight at 4°C. Biotinylated secondary antibodies were applied to sections at 1:200-600 and incubated for 30 min at room temperature. Sections were incubated with ABC reagent, developed in diaminobenzidine (DAB) and counterstained with haematoxylin. Slides were finally dehydrated and mounted. For the evaluation of the intensity and DAB-positive proportion of FGFR1-4 and brachyury immunostaining, the following scale was employed: 0: < 5% staining; 1: weakly stained cytoplasm and/or nuclei (pale brown); 2: moderate stained cytoplasm and/or nuclei (brown); and 3: strongly stained cytoplasm and/or nuclei, (dark brown). Final scores were derived from the multiplication of extent by intensity.

### FGF1 stimulation and FGFR/MAPK kinase inhibitor treatments

H460, H226 or H441 stable transfected cells were seeded in RPMI 1640 medium with 10% FBS in 6-well plates (for protein extraction and cell growth assay) or in 24-well plates (for immunofluorescence staining and cell invasion assay). The next day, medium was removed and replaced with serum-free medium. Cells at about 70% confluence were serum-starved overnight. For inhibitor experiments, cells were treated for 1 h with the FGFR inhibitor PD 173074 and MAPK kinase inhibitor PD 184352 at indicated concentrations before stimulation with 10 ng/ml or 100 ng/ml for different time points depending on the experiment.

### Quantitative real-time PCR

Total RNA (1 μg) from tissues and cells was reverse transcribed (RT) using Omniscript RT kit (Qiagen, Valencia, CA), Oligo (dT) 12–18 Primer (Invitrogen, Carlsbad, CA), and RNase inhibitor (Promega, Madison, WI). Amplification reactions were performed in triplicate in Applied Biosystems 7500 Real-Time PCR System using SYBR Green PCR master Mix (Applied Biosystems, Foster City, CA). The primers used for real-time PCR were: brachyury (forward: 5′-AGACTGGAGAGTTGGG-3′ and reverse: 5′- CAGGTGGTCCACTCGGTACT-3′), FGFR1 (forward: 5′-CCTGGTGACAGAGGACAATG-3′ and reverse: 5′-AGATCCGGTCAAATAATGCC-3′), FGFR2 (forward: 5′-AACGGGAAGGAGTTTAAGCA-3′ and reverse: 5′-CTTGTCAGATGGGACCACAC-3′), FGFR3 (forward: 5′-AGGCCATCGGCATTGACA-3′ and reverse: 5′-GCATCGTCTTTCAGCATCTTCAC-3’), FGFR4 (forward: 5′-GCGTCCACCACATTGACTAC-3′ and reverse: 5′-GTGTGTACACCCGGTCAAAC-3′), E-cadherin (forward: 5′-CCCACCACGTACAAGGGTC-3′ and reverse: 5′-ATGCCATCGTTGTTCACTGGA-3′), Snail (forward: 5′-GAGGCGGTGGCAGACTAG-3 and reverse: 5′-GACACATCGGTCAGACCAG-3′), β-catenin (forward: 5′-ACAAACTGTTTTGAAAATCCA-3′ and reverse: 5′-CGAGTCATTGCATACTGTCC-3′), fibronectin (forward: 5′-AAACCAATTCTTGGAGCAG G-3′ and reverse: 5′-CCATAAAGGGCAACCAAGAG-3′) and GAPDH (forward: 5′-CATGAGAAGTATGACAA CAGCCT-3′ and reverse: 5′-AGTCCTTCCACGATACCA AAGT-3′). All primers were synthesized by Integrated DNA Technologies (Coralville, IA). Each assay included a standard curve of five serial dilutions to quantify gene expressions in samples. Data were normalized to GAPDH and presents relative to control.

### Western blot assay

Cells were lysed in ice-cold lysis buffer (25 mM Tris-HCl, 150 mM NaCl, 1% Triton X-100, 0.1 mg/ml phenylmethanesulfonyl fluoride) with protease inhibitor cocktail and phosphatase inhibitor cocktail. Protein extracts were electrophoresed by 12.5% sodium dodecyl sulfate polyacrylamide gel electrophoresis and transferred to a nitrocellulose membrane. After blocking with 5% non-fat dry milk, the membrane was washed three times with Tris-buffered saline/Tween-20 and incubated with the primary antibody diluted in 3% BSA or 5% milk at 4°C overnight. The blot was washed in Tris-buffered saline/Tween-20 and incubated for 1h with a horseradish peroxidase-conjugated secondary antibody diluted 1:2000 for goat anti-rabbit (Cell signaling, Danvers, MA) or 1: 3000 for donkey anti-goat (Promega, Madison, WI). The signal was detected using the ECL Prime Western Blotting Detection reagent (GE Healthcare Bio-Sciences Corp, Pittsburgh, PA). Data are representative of three independent experiments.

### Immunofluorescence staining

H460, H226 or H441 cells stably transfected with pcDNA-FGFR1 were plated on coverslips in a 24-well plate containing RPMI 1640 medium with 10% FBS. Cells were transfected with control siRNA or FGFR1 siRNA or starved overnight and treated with FGF1 (10 ng/ml) for 24 h followed by PD 184352 (1 μM) treatment for 1 h. Cells were then fixed with 10% formalin for 15 min. Coverslips were rinsed with PBS and permeabilized with 0.2% Triton X-100 for 20 min, then washed three times with PBS and incubated with relevant primary antibody [anti-phosphorylated ERK 1/2 (1:200) and anti-brachyury antibodies (1:100) or anti-E-cadherin (1:100),anti-β-catenin (1:100), anti-Snail (1:100) and anti-fibronectin (1:100)] for 1 h. After washing with PBS, cells were incubated with a secondary antibody Alexa Fluor^®^ 488 AffiniPure Fab fragment goat anti-rabbit IgG (1:50) and/or Rhodamine Red™-X (RRX) AffiniPure F (ab′)_2_ fragment donkey anti-goat IgG (1:50) for 1 hour along with 4′,-6-diamidino-2-phenylindole (30 nM) for 15 min in the dark and washed three times with PBS. Cell images were captured under a fluorescent microscope (Olympus IX70) with a digital camera and processed using ImagePro^®^ Premier 9.1 software. Data are representative of three independent experiments.

### Cell growth assay

Stably transfected H460, H226, H441 or H358 cells were plated in 96-well plates at a density of 2 × 10^3^ cells per well in 100 μl of medium. After treatment with FGF1, PD 173074, PD 184352 and/or transfected with brachyury shRNA or scramble shRNA, cell growth was measured by MTS using a CellTiter 96^®^ AQueous One solution (Promega, Madison, WI) based on the manufacturer’s protocol. Data represent the mean absorbance of 4 or 5 wells and presents relative to control.

### Cell invasion assay

Cells (2.0 × 10^5^) were plated into the upper chambers of a BD Biocoat Matrigel Invasion Chambers (BD Bioscience, Bedford, MA) in serum-free media. PD 184352 (1 μM) was added to cells 1.5 h after plating and cells allowed to invade into the bottom chamber containing 10% FBS or 10 ng/ml FGF1. Chambers were then placed in 5% CO_2_ at 37°C for 24 h or 48 h. Non-invading cells were removed by scrubbing with a cotton tipped swab. The membrane was placed in 500 μl of methanol at room temperature for 10 min and then transferred to 500 μl of 1% crystal violet for 15 min to stain. Numbers of cells invaded to the underside of the membrane were scored from five microscope fields. The assays were performed in duplicate and repeated 4 times.

### Xenograft mouse studies

H460 and H441 human lung cancer cells were injected subcutaneously into the flank of 8-week-old female NCr/Nu nude mice (Charles River) (4 × 10^5^ cells per injection). Tumors were allowed to establish for 7 days, size matched, and allocated to groups of five animals. Treatment was by daily intraperitoneal injection with vehicle (5% DMSO, 95% water), 50 mg/kg PD 170374, 100 mg/kg PD 184352, or both for 12 d. Tumor size was determined by electronic caliper measurements of tumor length and width, and volume was calculated as volume = (length × width^2^)/2. All procedures using these animals were approved by the Institutional Animal Care and Use Committee of Wake Forest University and conducted in accordance with federal, state and institutional guidelines. The facilities and animal resources program of Wake Forest University are fully accredited by the Association for Assessment and Accreditation of Laboratory Animal Care (AAALAC).

### Statistical analysis

Data are expressed as means ± SD of at least triplicate measurements or SEM of at least three independent experiments. Statistical analysis was performed by SPSS V.10.0 for Windows using one-way analysis of variance with Bonferroni post-hoc test. *P* < 0.05 was considered statistically significant.
